# Electrodeposition of Copper and Brass Coatings with Olive-Like Structure

**DOI:** 10.3390/ma14071762

**Published:** 2021-04-02

**Authors:** Artur Maciej, Natalia Łatanik, Maciej Sowa, Izabela Matuła, Wojciech Simka

**Affiliations:** 1 Department of Inorganic Chemistry, Analytical Chemistry and Electrochemistry, Faculty of Chemistry, Silesian University of Technology, B. Krzywoustego Str. 6, 44-100 Gliwice, Poland; natalia.k.latanik@gmail.com (N.Ł.); maciej.sowa@polsl.pl (M.S.); wojciech.simka@polsl.pl (W.S.); 2 Faculty of Science and Technology, Institute of Materials Engineering, University of Silesia in Katowice, 75 Pułku Piechoty 1A, 41-500 Chorzów, Poland; imatula@us.edu.pl

**Keywords:** brass coating, Cu-Zn alloy coating, electrodeposition, non-cyanide bath, ionic liquids, 1-ethyl-3-methylimidazolium acetate, olive-like structure

## Abstract

One method of creating a brass coating is through electrodeposition, which is most often completed in cyanide galvanic baths. Due to their toxicity, many investigations focused on the development of more environmentally friendly alternatives. The purpose of the study was to explore a new generation of non-aqueous cyanide-free baths based on 1-ethyl-3-methylimidazolium acetate ionic liquids. The study involved the formation of copper, zinc, and brass coatings. The influence of the bath composition, cathodic current density, and temperature was determined. The obtained coatings were characterized in terms of their morphology, chemical composition, phase composition, roughness, and corrosion resistance. It was found that the structure of the obtained coatings is strongly dependent on the process parameters. The three main structure types observed were as follows: fine-grained, porous, and olive-like. To the best knowledge of the authors, it is the first time the olive-like structure was observed in the case of an electrodeposited coating. The Cu-Zn coatings consisted of 19–96 at. % copper and exhibited relatively good corrosion resistance. A significant improvement of corrosion properties was found in the case of copper and brass coatings with the olive-like structure.

## 1. Introduction

The most popular method to produce alloys is based on melting and casting the components of the alloy; however, it is not the only technique used to obtain alloys. Alloys may also be formed by other methods, e.g., sintering of metallic powders [1], diffusion saturation [2], and ion implantation [3]. Because of the high energy consumption, these methods are relatively costly, so they are only used in some specific cases where the conventional method is not economically justified, e.g., formation of alloys of refractory metals (W, Mo, Ta). Physical vapor deposition (PVD) and chemical vapor deposition (CVD) methods can be also used for formation of some specific alloys, e.g., high-entropy alloys [4] or diffusion barriers [5]. Moreover, alloys may also be formed during the process of electrodeposition, which is particularly interesting if the alloy is to be used as a coating. Electrodeposition of alloy coatings has many advantages, which are not encountered in the coatings obtained by other methods. First, the process is conducted at a relatively low temperature (up to several dozen °C), avoiding high costs of heating and melting the metals. The chemical composition, as well as the structure of the electrodeposited alloys, may be easily modified by galvanic bath composition or process parameters (e.g., current density). The method allows the formation of some phases which are impossible or hard to obtain by metallurgical methods. The thickness of the electrodeposited coatings is also uniform and easy to adjust. Moreover, the alloys obtained by electrodeposition are characterized by lower porosities relative to cast alloys or alloys formed by sintering of metallic powders [6]. The most popular and effective method of copper-zinc alloy (brass) coating formation is the electrodeposition in a cyanide solution. The galvanic baths have a high throwing power, the quality of the coatings is very good, and the current efficiency of the process is relatively high. Despite the high toxicity of cyanides and the strict maintenance control, the baths have been widely used in the production of brass alloy coatings [7]. There are many reports describing a possibility of the electrodeposition of Cu-Zn alloys in cyanide-free baths, e.g., pyrophosphate [8,9], citrate [10], oxalate [11], tartrate [12], ethylenediaminetetraacetic acid (EDTA) [13], triethanolamine [14], ammonia [15], glucoheptonate [16], glutamate [17], and glycinate [18] baths. The electrodeposited brass may be used as a matrix in the brass-composite coatings. These materials have been found to have improved properties, as compared to their non-composite counterparts, such as enhanced tribological properties in the case of graphite-brass coating [19] and boosted scratch and corrosion resistance in the case of Cu_5_Zn_8_-brass intermetallic composite coating [20].

To address the need for more alternatives to the cyanide-based baths, a new generation of galvanic baths based on deep eutectic solvents (DES) or ionic liquids (IL) has been developed. Most often, these methods have many advantages over aforementioned bath compositions such as being considered green, non-flammable solvents, as well as typically having low vapor pressures, high thermal stability, and a wide electrochemical window [21,22]. The non-aqueous electrolytes allow to form many popular metallic coatings like zinc [23], tin [24], nickel [25], chrome [26], as well as some important metals and their alloys which cannot be electrodeposited from aqueous baths like titanium [27], aluminum [28], and magnesium [29]. The most popular DESs used for electrodeposition are based on choline salt eutectic mixtures [30], mainly with urea [31], thiourea [32], ethylene glycol [33], or salts being the source of the deposited metals [34]. Moreover, some chloride-free solutions, e.g., based on choline dihydrogencitrate [35] or choline acetate [36] can be used for metals and alloys electrodeposition. Any of these options may be successfully used as a cyanide-free galvanic bath for Cu-Zn alloy coatings. Xie et al. [37] investigated the electrodeposition of Zn and Cu-Zn coatings from a choline chloride/urea-based DES with ZnO and CuO precursors. The conditions for uniform, dense, and compact coatings were determined. Moreover, it was found that the compact, flat, and fine particles ensured improved corrosion resistance in a sodium chloride solution. Electrodeposition of brass coatings was also investigated by Fricoteaux et al. [38] using a 1-butyl-1-methylpyrrolidinium bis(trifluoromethylsulfonyl)imide IL. The authors reported that the electrodeposition of Zn, Cu, and Cu-Zn coatings is possible from ILs without the use of chloride anions. The obtained layers had different morphologies and phase compositions corresponding to the mixtures of different Cu-Zn phases that were obtained from a range of applied potentials. It was found that between a specific range of potentials (from −1.6 to 2.25 V/Ag), the zinc content in the alloy decreased and the coatings became amorphous. Zhang et al. [39] also studied the Cu-Zn electrodeposition coating process when using 1-ethyl-3-methylimidazolium trifluoromethylsulfonate IL and ethanol mixtures to replace the highly-poisonous cyanide zincate process. The Zn content in the obtained coatings was up to 30 at. %. It was reported that under optimal conditions, the cauliflower-shaped Cu_5_Zn_8_ alloy displayed a significant improvement in corrosion resistance when electrodeposited on a metallic substrate. Based on our previous work [40], it was found that the use of non-aqueous galvanic baths based on acetate anion allows for electrodeposition of good-quality coatings of zinc, copper, and Cu-Zn alloys. So, the authors of this paper have considered using galvanic baths based on 1-ethyl-3-methylimidazolium acetate ([EMIM][Ac]) IL to eliminate the use of cyanide in aqueous baths for the electrodeposition of brass coatings. It should be added that the ionic liquid is commercially available which is an advantage, in the prospect of its potential application.

## 2. Materials and Methods

### 2.1. Electrodeposition of Zn, Cu, and Cu-Zn Coatings on Steel

Copper (Cu), zinc (Zn), and Cu-Zn alloy coatings were electrodeposited on S235JR 100 carbon steel substrate (SAG Sp. z o.o., Katowice, Poland) whose chemical composition is as follows: ≤0.22% C; ≤1.1% Mn; ≤0.35% Si; ≤0.05% P; ≤0.05% S; ≤0.30% Cr; ≤0.30% Ni; 0.30% Cu; 0.02% Al and the remain is Fe. Electrolytes based on mixtures of 1-ethyl-3-methylimidazolium acetate ([EMIM][Ac]) and acetate(s) of appropriate metal(s), i.e., copper acetate (CuAc: 5–80 g·L^−1^) for copper electrodeposition, zinc acetate (ZnAc: 5–800 g·L^−1^) for zinc electrodeposition or both of the salts (CuAc and ZnAc) for the brass coatings have been used (Table 1). Preparation of the electrolytes is based on the addition of portions of the acetate(s) of appropriate metal(s) to the liquid [EMIM][Ac] and mixing with a magnetic stirrer (200 rpm; MR Hei-Tec, Heidolph, Schwabach, Germany). The purity and suppliers of the acetates are as follows: [EMIM][Ac] (≥95%/HPLC), Sigma–Aldrich Chemie GmbH, Schnelldorf, Germany; ZnAc (pure), POCh, Gliwice, Poland; CuAc (pure), POCh, Gliwice, Poland.

The electrodeposition of copper and zinc was conducted at different cathodic current density values (j_c_) of 0.75; 1.5; 3; 6; 12; 24; 48 mA·cm^−2^ and at operating temperatures of 25; 50; 75; and 100 °C. The range of the conditions has been selected based on our experience acquired during our previous work [40]. The steel samples were used as the cathode (substrate), and platinum plates (Mennica Polska S.A., Warszawa, Poland) were used as anodes. The geometrical area of the working electrode was 2 cm^2^—in the case of standard samples and 4 cm^2^—in the case of samples used for the current efficiency determination. A pair of platinum plates of the same dimensions as the cathode was stacked in parallel to the steel substrate.

The parameters of the Cu-Zn electrodeposition coatings were selected based on the studies of copper and zinc formation in the acetate baths. The four galvanic baths, based on 1-ethyl-3-methylimidazolium acetate, were prepared for the investigation; their composition is presented in Table 1. The process was conducted in the predetermined range of cathodic current density (1.5; 3; 6; and 12 mA·cm^−2^) and at a favourable temperature (100 °C). The electrodeposition period varied between 60 min (for the lowest j_c_ = 0.75 mA·cm^−2^) and 56 s (for the highest j_c_ = 48 mA·cm^−2^) in order to ensure a constant electric charge per unit surface area for all specimens of 2.7 C·cm^−2^.

### 2.2. Characterization

The surface morphology was investigated by a Phenom ProX (Thermo Fisher Scientific, Waltham, MA, USA) scanning electron microscope (SEM) equipped with an energy dispersive X-ray spectroscopy (EDX, Thermo Fisher Scientific, Waltham, MA, USA) analyzer to determine the surface chemical composition. The roughness of the coatings was determined based on 3D surface reconstructions obtained by the SEM and served to generate microroughness profiles of the coatings. The profiles were used to calculate the arithmetic roughness coefficient (Ra) and the maximum height of the profile (Rz). The chemical compositions of the brass coatings were also analyzed by inductively coupled plasma optical emission spectroscopy (ICP-OES) by an ICP-OES Varian 710-ES spectrometer (Varian Inc., Hansen Way Palo Alto, CA, USA) after etching of the coatings in 10% HNO_3_ (prepared by dilution of 65% HNO_3_, pure for analysis, P.P.H. Stanlab Sp. z o.o., Lublin, Poland) at a temperature of 60 °C. Based on the mass growth of the sample which was determined by means of XS 105 Dual Range analytical balance (Mettler Toledo, Columbus, OH, USA) the current efficiency (CE) of the electrodeposition processes and thickness of the coatings were determined. Moreover, the electrodeposition rate, presented as the thickness growth per minute, was calculated. The phase composition was determined by an X’Pert PW3040/60 (Grazing incidence X-ray diffraction—GIXD) X-ray diffractometer (Philips, Eindhoven, The Netherlands). The GIXD diffraction patterns were registered in 2θ range from 10° to 120° and 0.05° step for the incident angle α: 0.25; 0.50; 1.00; 2.50; and 5.00 degrees, respectively. In order to maintain comparable intensities of the diffraction lines, the conditions for collecting patterns (step and counting time) were properly adjusted. GIXD uses small incident angles (α) for the incident X-ray beam, so that it is used to study surface layers as the beam penetration is limited. The corrosion resistance studies of the electrodeposited coatings were carried out by the potentiodynamic Tafel method using an AUTOLAB PGSTAT100 (Metrohm, Utrecht, The Netherlands) potentiostat-galvanostat. The thickness of the studied coatings was 8 ± 2 μm. The corrosion measurements were carried out in a 5% solution of NaCl (prepared by dissolution of NaCl, pure for analysis, Chempur, Piekary Śląskie, Poland) at a temperature of 25 °C. The samples were stabilized at open circuit (up to 1 h) before the polarization and the open circuit potentials (E_OC_) were determined. The potentiodynamic measurements (linear sweep voltammetry—LSV) were carried at potentials between E_OC_ −150 mV and E_OC_ +150 mV with a scan rate of 0.1 mV·s^−1^. The obtained potentiodynamic curves were analyzed by NOVA 2.1. software (ver. 2.1., Metrohm, Utrecht, The Netherlands), which was able to obtain the corrosion properties of the studied systems, such as polarization resistance (R_p_), corrosion current density (j_corr_), and corrosion potential (E_corr_). A double-walled (thermostatic) electrochemical cell with a three-electrode configuration was used (BioLogic, Seyssinet-Pariset, France). A saturated calomel electrode (SCE, BioLogic, Seyssinet-Pariset, France) served as the reference electrode, and a platinum mesh (BioLogic, Seyssinet-Pariset, France) served as the counter electrode.

## 3. Results and Discussion

### 3.1. Surface Morphology—SEM Analysis

#### 3.1.1. Copper Coatings

**Figure 1:**
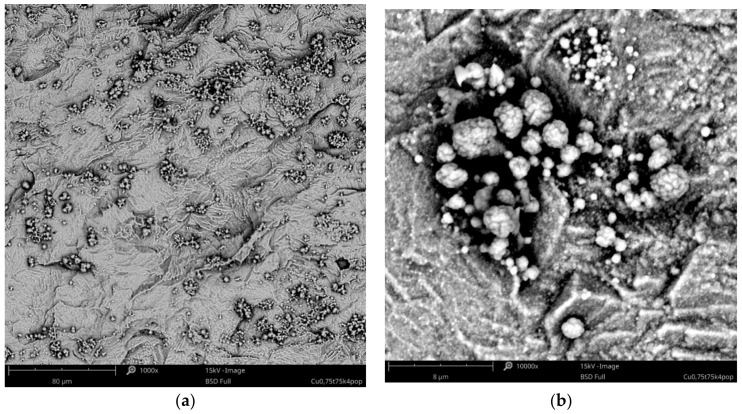
SEM images of Cu island electrodeposited in the bath with 20 g·L^–1^ of CuAc at a temperature of 75 °C (j_c_ = 0.75 mA·cm^−2^, t = 1 h) at low (**a**) and high (**b**) magnification.

**Figure 2:**
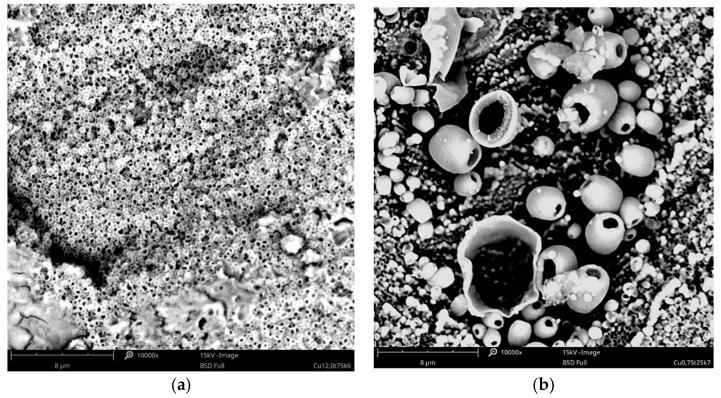
SEM images of the porous Cu coating electrodeposited in the bath with: (**a**) 30 g·L^−1^ of CuAc (T = 75 °C, j_c_ = 12 mA·cm^−2^, t = 7.5 min); (**b**) 35 g·L^−1^ of CuAc (T = 25 °C, j_c_ = 0.75 mA/cm^−2^, t = 1 h).

The effect of copper acetate concentration, temperature, and cathodic current density on the structure of the copper coatings was investigated. All parameters had an influence on the behavior of the coating forming process and each coating’s morphology. In the baths with low concentrations of copper salts (5–10 g·L^−1^), the electrodeposition of the coating was not possible. Electrodeposition of copper is only feasible in baths with relatively high concentrations of the metal, especially at elevated temperatures (75, 100 °C). Small amounts of copper in the form of islands were electrodeposited from the baths with a relatively low concentration of copper acetate: 20–30 g·L^−1^ (Figure 1). The observed type of crystallization occurred through Volmer–Weber island growth, which can result from the diffusion-limited regimes [41]. Some of the obtained coatings had very characteristic, porous structures, which are not typical for electrodeposited coatings. The specific process parameters led to the development of these structures. It was observed that the homogeneous, porous coatings with pores sizes less than 500 nm were obtained in a bath with 30 g·L^−1^ of CuAc at a temperature of 75 °C (Figure 2a). Other porous coatings were obtained in a bath containing 35 g·L^−1^ of CuAc at a temperature of 25 °C (Figure 2b).

To the best knowledge of the authors, it is the first time that the olive-like structure was observed in an electrodeposited coating. A similar structure has been seen by Rokosz et al. [42,43] and Komarova et al. [44] in the case of oxide coatings obtained by a plasma electrolytic oxidation (PEO) process of titanium, but the mechanism by which the structure forms was drastically different. The most probable mechanism of the olive-like structure generation describes the crystallization of the metal around a small hydrogen bubble that adheres to the surface during the formation. The hydrogen bubble can be formed during electrolysis because of residual water present in the bath from hydrated salts or from the surrounding air (in the present study, the galvanic bath was not protected against contact with air).

**Figure 3:**
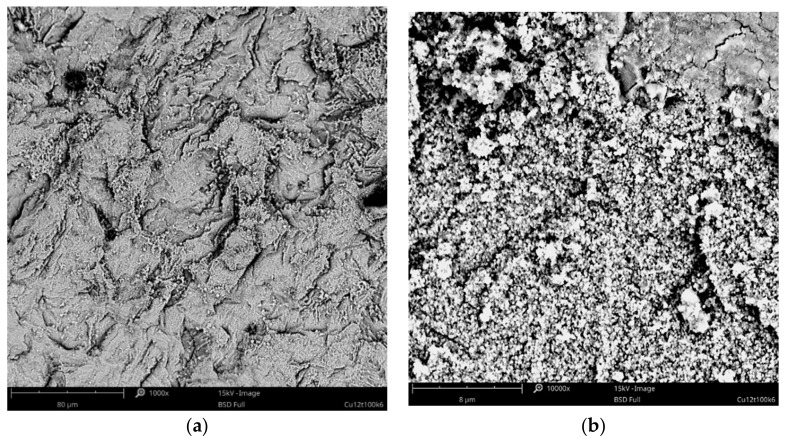
SEM images of the Cu coating electrodeposited in the bath with 30 g·L^−1^ of CuAc at a temperature of 100 °C (j_c_ = 12 mA cm^−2^, t = 7.5 min) at low (**a**) and high (**b**) magnification.

**Figure 4:**
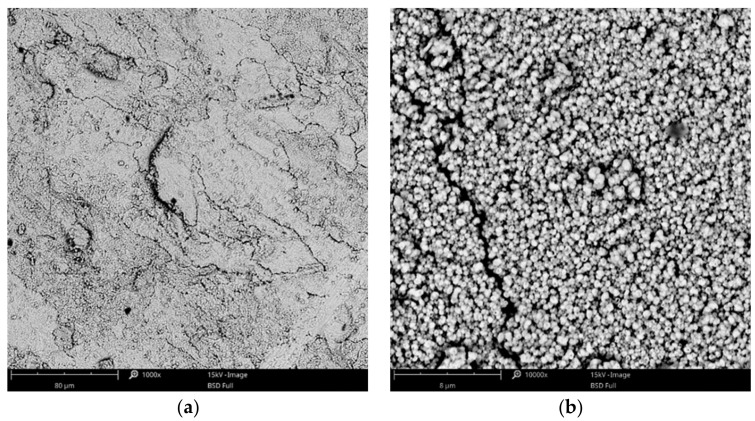
SEM images of the Cu coating electrodeposited in the bath with 60 g·L^−1^ of CuAc at a temperature of 75 °C (j_c_ = 12 mA·cm^−2^, t = 7.5 min) at low (**a**) and high (**b**) magnification.

Based on the preliminary results, subsequent experiments were focused on the process in baths with higher concentrations of copper acetate (35–60 g·L^−1^ of CuAc) and at the favorable temperatures of 75 and 100 °C. These parameters allowed for obtaining uniform and compact coatings, especially in the range of current density from 3 to 12 mA·cm^−2^. The surface morphologies of some exemplary coatings with fine-grained structures are presented in Figure 3 and Figure 4. The coatings obtained in the Cu-10 bath, with higher concentration of CuAc (80 g·L^−1^), had a powder-like structure so further increasing of copper acetate percentage was not reasonable.

#### 3.1.2. Zinc Coatings

**Figure 5:**
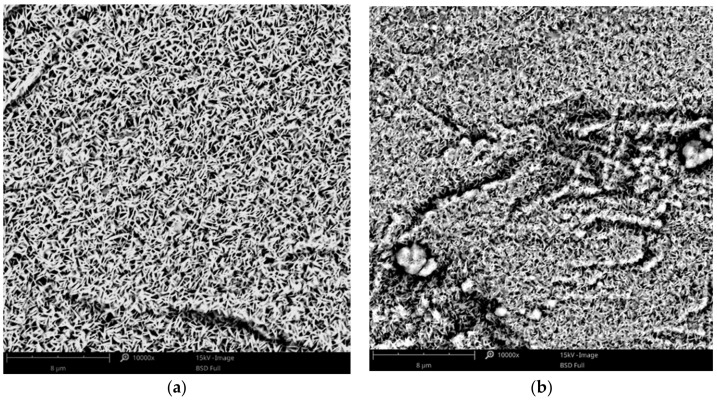
SEM images of the Zn coating electrodeposited in the bath with 430 g·L^−1^ of ZnAc at a temperature of: (**a**) 75 °C (j_c_ = 3 mA·cm^−2^, t = 15 min); (**b**) 100 °C (j_c_ = 24 mA·cm^−2^, t = 2 min).

**Figure 6:**
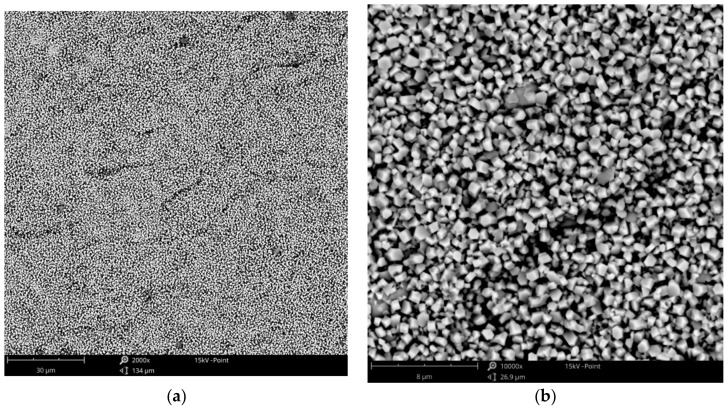
SEM images of the Zn coating electrodeposited in the bath with 800 g·L^−1^ of ZnAc at a temperature of 100 °C (j_c_ = 3 mA·cm^−2^, t = 15 min) at low (**a**) and high (**b**) magnification.

In the case of the zinc electrodeposition, the formation of coatings was observed only from the baths with relatively high zinc acetate concentrations (>430 g·L^−1^ of ZnAc). Zinc coatings were not possible in the baths with lower concentrations. The coatings deposited from the bath containing 430 g·L^−1^ of ZnAc had a characteristic, needle-shaped structure, and were not very tight nor compact (Figure 5). The coatings were also not macroscopically homogeneous. However, the increase in the zinc salt concentration to 600 or 800 g·L^−1^ ensured that the coatings were fine-grained as well as uniform, in the macro- and micro-scale (Figure 6). The best quality of the zinc coatings has been evidenced for cathodic current density values in the range of 1.5 to 6.0 mA·cm^−2^. In addition, electrolyte temperatures between 75 and 100 °C facilitated adequate deposits even at lower current densities, while at higher values, dull, poor quality coatings were observed.

#### 3.1.3. Cu-Zn Coatings

**Figure 7:**
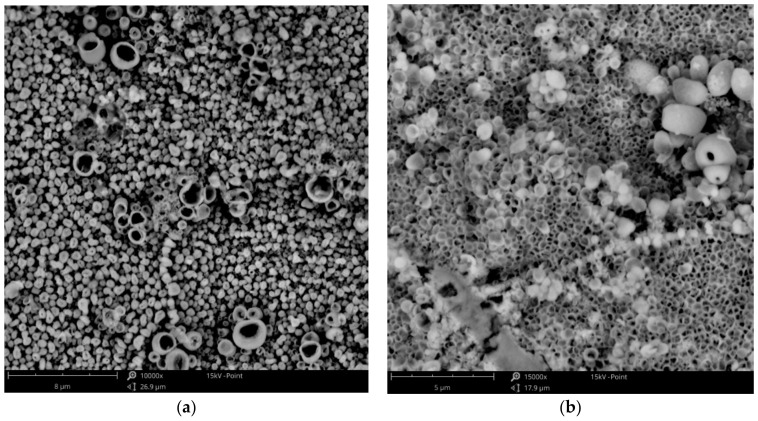
SEM images of the Cu-Zn coating electrodeposited in the CuZn-1 bath at a temperature of 100 °C, at a current density of: (**a**) j_c_ = 1.5 mA·cm^−2^; (**b**) j_c_ = 12 mA·cm^−2^.

The use of baths with lower concentrations of zinc (CuZn-1 and CuZn-2) resulted in the formation of homogeneous and porous coatings. The deposited layers obtained at low current densities had the characteristic olive-like structure (Figure 7a), similar to the one received in the case of the copper coating (Figure 2b). Both coatings were electrodeposited at low current densities (0.75 and 1.5 mA·cm^−2^), which suggests that the low deposition rate favors the olive-like structure formation. The increase in the current density to 12 mA·cm^−2^ led to a change in the structure. The typical olive-like structures were observed less often and had a different characteristic porous structure (Figure 7b). In the case of the coatings electrodeposited in the baths with higher concentration of zinc (CuZn-3 and CuZn-4 baths), the structure was mainly fine-grained, similar to the copper coating (Figure 4).

### 3.2. Chemical Composition of the Cu-Zn Coatings

**Figure 8:**
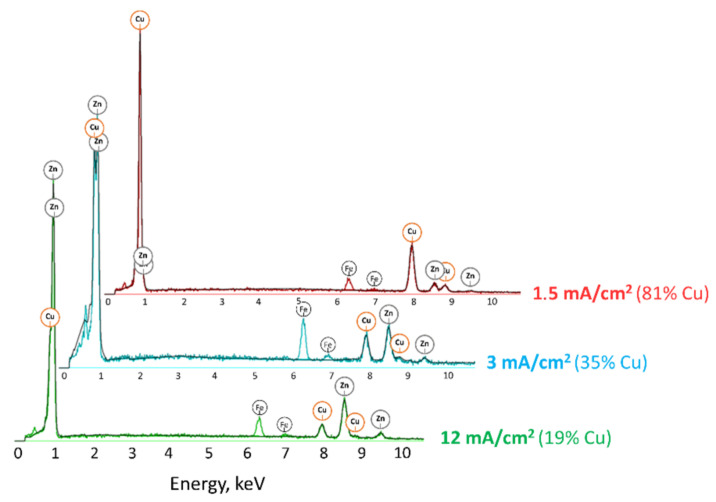
The EDX spectra of the brass coatings electrodeposited in the CuZn-4 bath (T = 100 °C).

The chemical composition of the brass coatings was dependent on the bath composition as well as the current density. It was analyzed by two methods: EDX and ICP-OES. The exemplary EDX spectra, which show the significant influence of current density on the composition of the alloy, are presented in the Figure 8.

**Figure 9:**
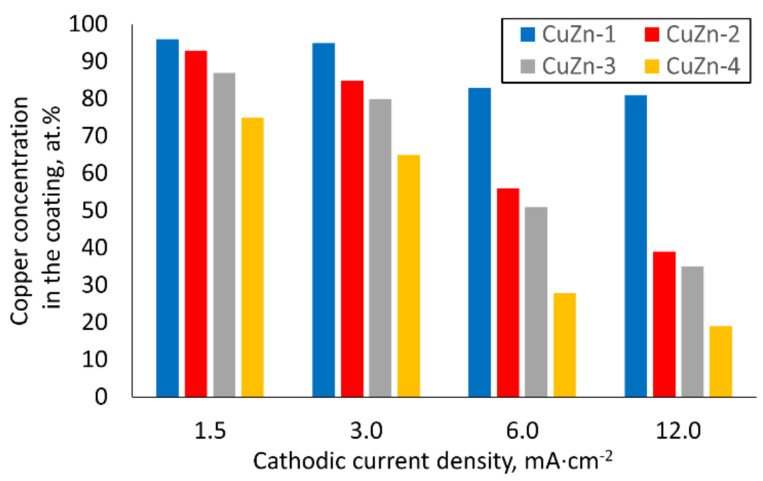
The bar chart showing chemical composition of the brass coatings (at. % of copper).

It was noted that in the case of all galvanic baths, the increase in current density caused a decrease in the amount of copper present in the alloy. Moreover, the concentration of zinc acetate in the bath also had a strong influence on the alloy composition. The amount of zinc in the alloy increased with increasing amounts of ZnAc in the bath. The comparison of the chemical composition of the received brass alloys is presented in Figure 9. The results obtained by ICP-OES are nearly identical to those obtained by EDX analysis; the differences were less than 2 at. %. The highest concentration of copper (96 at. %) was detected in the case of the alloy deposited in the CuZn-1 bath at a current density of 1.5 mA·cm^−2^ and the lowest percentage of copper (19 at. %) was detected for the alloy formed in the CuZn-4 bath at a current density of 12 mA·cm^−2^. Therefore, the composition of the brass coatings may be relatively easily varied by the use of two parameters (i.e., j_c_ and bath composition) over a wide range of chemical compositions of the alloy.

**Figure 10:**
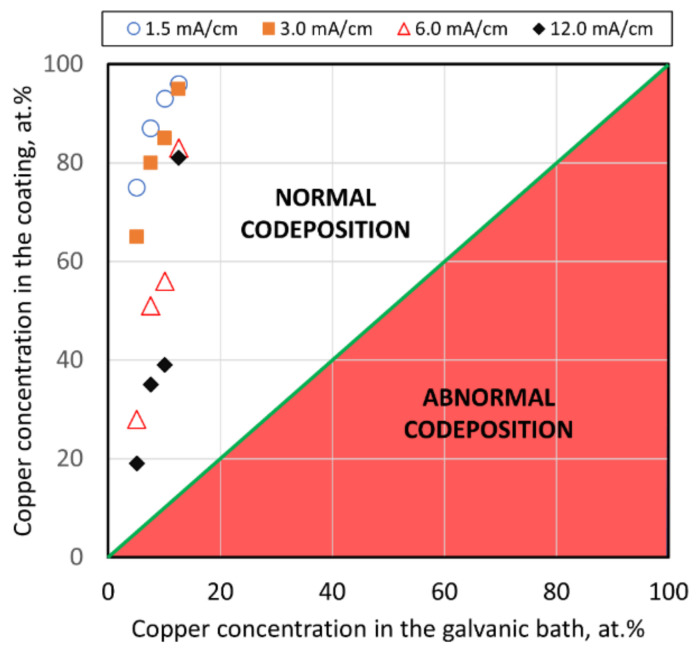
Graphical presentation of the influence of the galvanic bath composition and current density on the type of codeposition according to Brenner’s classification (green line refers to regular codeposition).

The correlation between chemical composition of the alloys and the galvanic baths which were used for their formation led to the classification of each specific type of electrodeposition processes. According to the Brenner’s classification [45], in the studied range of parameters, the electrodeposition can be assigned as a normal type of codeposition. It means that copper, which is a more noble metal than zinc, deposits preferentially and the content of copper in the alloy is higher than in the plating solution. The correlation is presented in Figure 10.

Normal codeposition is also typical in the case of electrodeposition of brass coatings from cyanide baths, but it should be noted that abnormal codeposition with underpotential deposition of zinc is also possible [46].

### 3.3. Current Efficiency and Electrodeposition Rate

**Figure 11:**
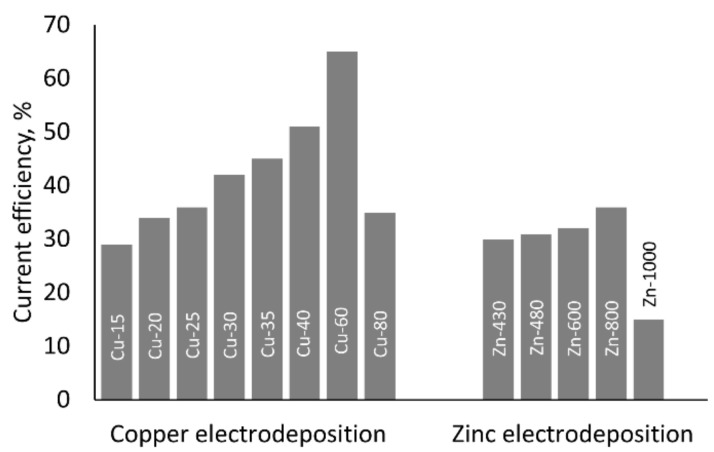
The effect of metal acetate (CuAc or ZnAc) concentration on current efficiency of copper and zinc electrodeposition at 100 °C, conducted at j_c_ = 3 mA·cm^2^.

The described effect of metal acetates concentrations in the galvanic baths on the quality of the obtained coatings (Section 3.1) was in good accordance with changes of current efficiency of the process. Figure 11 presents an example of the dependence of the current efficiency vs. the metallic salt content in the case of a constant applied cathodic current density of 3 mA·cm^−2^. It was observed that usually, an increase in the metallic salt concentration in the electrolyte facilitated higher current efficiency (CE) up to a certain value, followed by a significant decrease for the highest ones. Therefore, in the case of copper coating, the highest value of CE, of 65%, was obtained in the Cu-60 bath at 100 °C. The current efficiency of zinc electrodeposition was relatively low. At the optimum temperature of 100 °C and j_c_ = 3 mA·cm^−2^, CE increased from 30 up to 36% as the ZnAc concentration was raised from 480 to 800 g·L^−1^, respectively.

**Figure 12:**
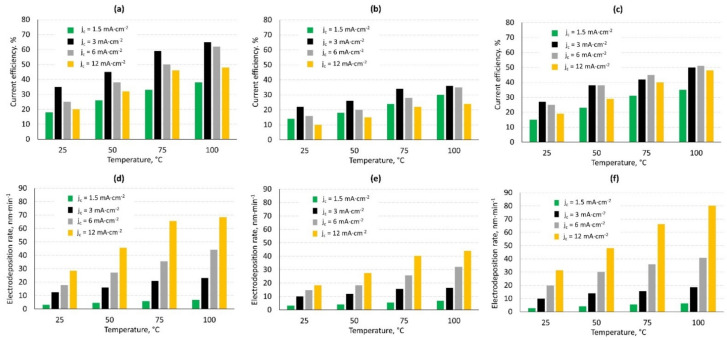
The effect of cathodic current density and temperature on the current efficiency (**a**–**c**) and electrodeposition rate (**d**–**f**) of copper from Cu-60 bath (**a**,**d**), zinc from Zn-800 bath (**b**,**e**) and CuZn alloy coatings from CuZn-3 bath (**c**,**f**).

Based on the results, the studies of influence of temperature and current density were conducted for the baths with the highest CE (Cu-60 bath, Zn-800 bath). The case of combination of the two baths used for Cu-Zn alloy coatings formation (CuZn-3 bath) was also studied. Moreover, the electrodeposition rates for the processes were determined (Figure 12). Regardless of the type of bath, the influence of temperature and current density was similar. In all of the cases, the current efficiency increased with the increase in temperature and the highest values were observed for the processes performed at 100 °C. In compliance with the results presented in Figure 11, the electrodeposition of copper was characterized by the higher current efficiency than the zinc deposition. The thickness of the obtained copper coatings was in the range from 1.9 to 7.0 μm, depending on the current efficiency whereas the thickness of the zinc coatings was in the range of 1.3 to 4.9 μm. The current efficiency of CuZn alloy coatings deposition was intermediate and it was in the range of 15% (T = 25 °C and j_c_ = 1.5 mA·cm^−2^) to 51% (T = 100 °C and j_c_ = 6 mA·cm^−2^). The determined CE values are lower than in the case of brass deposition from aqueous cyanide baths where the current density achieves 65–75%. Thicknesses of the alloy coatings were in the range of 1.6 to 6.1 μm. Because of the necessity of the use of relatively low current density, the electrodeposition rate of the processes was not high. Regardless of type of electrodeposited coatings, the highest values were observed for the coatings electrodeposited at the highest current density (12 mA·cm^−2^). The lowest electrodeposition rate was marked in the case of the zinc coatings formation (3.2–44 nm·min^−1^). The rate of copper coatings deposition was higher; it was in the range of 3.2 to 68.4 nm·min^−1^. The highest electrodeposition rate equals to 80.1 nm·min^−1^ was determined for CuZn coating, formed at 100 °C and j_c_ = 12 mA·cm^−2^_._

### 3.4. Phase Composition of the Coatings

**Figure 13:**
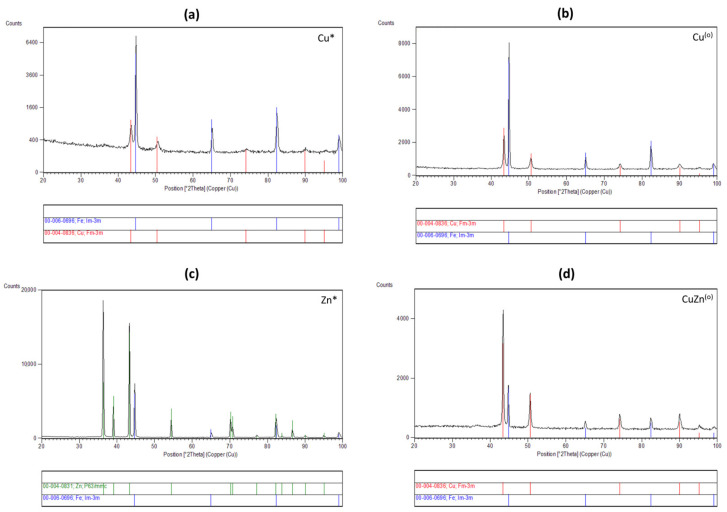
The X-ray diffraction patterns of: (**a**) fine-grained copper coating; (**b**) olive-like copper coating; (**c**) fine-grained zinc coating; (**d**) olive-like structured CuZn coating (4 at. % of Zn). *—fine-grained structure; ^(o)^—olive-like structure.

The recorded diffraction patterns (α = 0.50) of the studied samples with Cu, Zn, and Zn-Cu alloys are presented in Figure 13. The diffraction patterns signify that the layer has a crystalline character. Qualitative phase analysis of samples shows that the layers are composed of face-centred cubic Cu (International Centre for Diffraction, Data Powder Diffraction File, ICDD PDF 00-004-0836) or hexagonal close-packed Zn (ICDD PDF 00-004-0831) phases for Cu, Zn-Cu and Zn layer, respectively. Furthermore, the substrate as Fe phase is observed (ICDD PDF 00-006-0696). The studies received an interesting information showing the same structure (Fm-3m) for fine-grained copper coatings (Figure 13a) and olive-like copper coating (Figure 13b). Moreover, the X-ray pattern obtained for CuZn alloy coating (4 at. % of zinc) with olive-like structure (Figure 13d) indicated that the layer also has the cubic structure which corresponds to solid solution zinc in copper (α-brass). It is a typical single phase brass structure for CuZn alloys containing up to 32% of zinc. The obtained zinc coatings have the hexagonal structure, which is also typical for electrodeposited zinc coatings from aqueous solutions.

### 3.5. Roughness of the Olive-Like-Structured Brass Coatings

**Figure 14:**
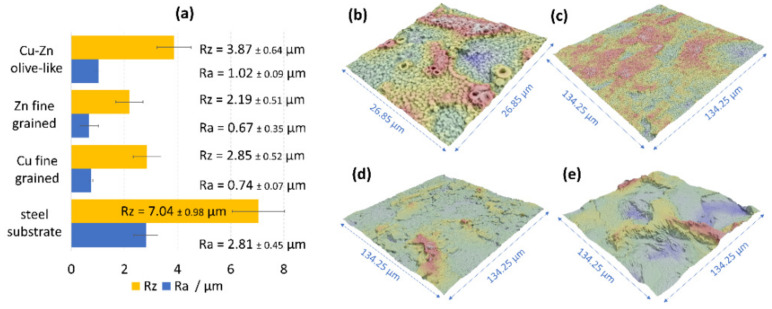
Roughness results (**a**) obtained on the basis of the 3D surface reconstructions of: (**b**) brass coating of the olive-like structure; (**c**) fine-grained copper coating; (**d**) fine-grained zinc coating; (**e**) etched steel substrate.

Based on the 3D surface reconstructions, the characteristic parameters (R_a_, R_z_) for the brass coating with the olive-like structure, as well as the fine-grained copper and zinc coatings, were determined (Figure 14). Despite the high porosity of the brass coatings, the roughness of the ones obtained in this study were significantly lower than the roughness of the etched S235JR steel, which was a substrate in the process. The arithmetic roughness coefficient (R_a_) for the steel was 2.85 ± 0.45 μm, whereas the R_a_ for the olive-like structured brass was 1.02 ± 0.45 μm. However, the fine-grained coatings of copper and zinc had much lower roughness: 0.76 ± 0.07 μm and 0.67 ± 0.07 μm, respectively. The results obtained for the three types of coatings showed that the acetate galvanic baths based on 1-ethyl-3-methylimidazolium acetate led to deposition of coatings with the diminished surface roughness without the use of any additives.

### 3.6. Corrosion Resistance of the Coatings

**Figure 15:**
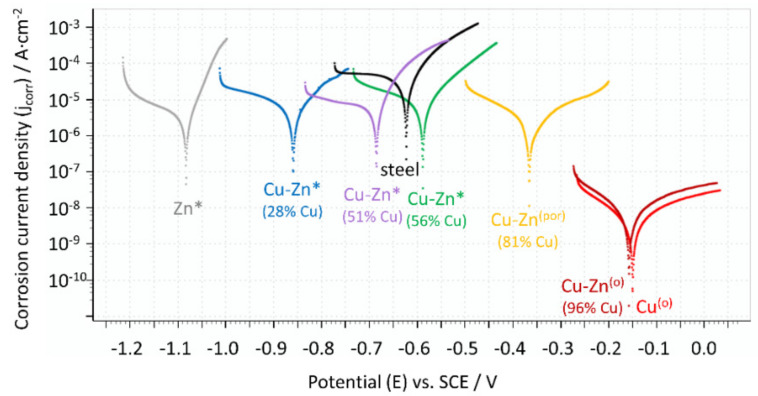
Potentiodynamic curves for the steel substrate as well as for the selected coatings (Cu, Zn, Cu-Zn) with different structure: *—fine grained coatings; (por)—porous coating; ^(o)^—coatings with the olive-like structure.

Corrosion resistance studies were focused on the selected coatings, which had different chemical compositions and different structures. Moreover, the measurements were also done for the steel substrate for the sake of comparison. The obtained potentiodynamic curves are presented in the Figure 15, and all parameters that defined the corrosion resistance were arranged in Table 2.

The most electropositive corrosion potential was determined for the copper coating (E_corr_ = −0.148 V) and the most electronegative was for the zinc coating (E_corr_ = −1.082 V), which is in good accordance with their position in the electrochemical series. The brass coatings had the intermediate values of corrosion potential, which increased with the increasing copper concentration in the alloy. A similar relationship has been observed by El-Sherif et al. [47], who studied the electrochemical behavior of brasses with varied zinc percentages.

The carbon steel substrate corrodes relatively easily and requires proper corrosion protection. The weak corrosion resistance of the material was confirmed by the potentiodynamic studies in which the highest corrosion current density (j_corr_ = 27.8 μA·cm^−2^) and the lowest polarization resistance (R_p_ = 0.9 kΩ·cm^2^) were determined. The values of corrosion potential for some of the obtained coatings were more electronegative than the E_corr_ value of the steel (E_corr_ = 0.621 V), meaning the coatings exhibited anodic character. They are zinc coatings and brass coatings with a relatively low percentage of the more noble metal (up to 51 at. % of copper). The copper coating, as well as the brass coating with a concentration of copper above 56 at. %, had a more electropositive corrosion potential than steel, so they were cathodic type coatings in the chloride solution.

It was found that the structure of the studied coatings had significant effects on the corrosion properties of the coating. All fine-grained coatings were characterized by a good corrosion resistance, regardless of their composition. In this group of coatings, the corrosion current density values were between 5.2 and 6.5 μA·cm^−2^ and polarization resistance values were between 3.67 and 4.79 kΩ·cm^2^. The brass coating (81 at. % of Cu) with the porous structure, presented in Figure 7b, had slightly better anticorrosion properties (j_corr_ = 1.97 μA·cm^−2^, R_p_ = 13.65 kΩ·cm^2^) than the fine-grained coatings. The results obtained for Cu and CuZn coatings with the olive-like structures were surprising because the corrosion parameters (j_corr_, R_p_) were three orders of magnitude different than those of the fine-grained coatings, which indicates extremely high corrosion resistance. Despite of the same chemical and phase compositions of the fine-grained copper coating and the olive-like copper coating there is a significant difference in terms of their corrosion resistance. It suggests that the morphology of the coatings plays an important role in terms of their corrosion properties. The most probable reason for the phenomenon is the hindered penetration of the corrosion medium into the inside part of the olive-like pores ensured by their very characteristic structure. After the immersion, the pores were still saturated by air and the corrosive solution had no direct contact with the surface of the porous coating. Moreover, the hypothesis could be lent credence by the fact that the improved porous coating (with 81 at% of Cu) had a mainly open-pore structure with some olive-like structures (lone olives, also noticeable in Figure 7b). A similar explanation which connects the improved corrosion resistance of porous materials with their structure has been presented by Seath et al. [48], who found that for unsintered porous titanium, the corrosion resistance increases with porosity.

## 4. Conclusions

The galvanic baths based on 1-ethyl-3-methylimidazolium acetate ionic liquid allow for the electrodeposition of copper, zinc, and brass coatings. The structure of the obtained coatings is strongly dependent on the process parameters. The three main structures observed were fine-grained, porous, and olive-like. The composition of the brass coatings could be varied by cathodic current density, as well as bath composition over a wide range of chemical compositions of the alloy. The parameters led to alloys with 19–96 at. % copper. Brass electrodeposition occurs as a normal type of codeposition, according to Brenner’s classification. The electrodeposited coatings were characterized by lower roughness than the steel substrate. All metallic and alloy coatings had good corrosion resistance, which were found to be dependent on their structure. Substantial improvement in corrosion properties was found in the case of copper and brass coatings with olive-like structures. The proposed procedure of electrodeposition allows for the formation of porous copper and brass coatings, which could serve as alternatives to other methods, e.g., powder sintering. Electrodeposition has the potential to be used in many applications such as for supports for catalysts and for the production of active electrodes for electrocatalysis. Moreover, the porous coatings may be modified by incorporating corrosion inhibitors or other substances in order to improve their functional properties.

## Figures and Tables

**Table 1:** Chemical composition of the galvanic baths for Cu, Zn, Cu-Zn coatings electrodeposition.

Desirable Type of Coating	Bath Symbol	Component			at. % of Cu in Bath
		[EMIM][Ac]	CuAc, g·L^−1^	ZnAc, g·L^−1^	
Copper coating	Cu-5	Basic electrolyte (solvent)	5	-	100
	Cu-10		10	-	
	Cu-20		20	-	
	Cu-25		25	-	
	Cu-30		30	-	
	Cu-35		35	-	
	Cu-40		40	-	
	Cu-60		60	-	
	Cu-80		80	-	
Zinc coating	Zn-0 group	Basic electrolyte (solvent)	-	5–370 (no deposit)	0
	Zn-430		-	430	
	Zn-480		-	480	
	Zn-600		-	600	
	Zn-800		-	800	
	Zn-1000		-	1000	
Cu-Zn alloy coating	CuZn-1	Basic electrolyte (solvent)	60	480	12.6
	CuZn-2		60	600	10.1
	CuZn-3		60	800	7.5
	Cu-Zn-4		60	1000	6.1

**Table 2:** Summary of the results obtained by the Tafel potentiodynamic method.

Coating Type	at. % of Cu in Coating	Structure of Coating	E_corr_ vs. SCE (V)	j_corr_ (μA·cm^−2^)	R_p_ (kΩ·cm^2^)
Cu	100	olive-like	−0.148	3.1·10^−3^	4726.8
Cu	100	fine-grained	−0.146	5.22	4.79
Cu-Zn	96	olive-like	−0.156	5.3·10^−3^	4359.0
Cu-Zn	81	porous	−0.364	1.97	13.65
Cu-Zn	56	fine-grained	−0.587	6.50	3.67
Cu-Zn	51	fine-grained	−0.684	5.54	3.76
Cu-Zn	28	fine-grained	−0.858	5.48	4.58
Zn	0	fine-grained	−1.082	5.60	3.97
Steel (substrate)	-	-	−0.621	27.8	0.90

## Data Availability

Data are contained within the article.
